# Design of Trench MIS Field Plate Structure for Edge Termination of GaN Vertical PN Diode

**DOI:** 10.3390/mi14112005

**Published:** 2023-10-28

**Authors:** Sung-Hoon Lee, Ho-Young Cha

**Affiliations:** School of Electronic and Electrical Engineering, Hongik University, 94 Wausan-ro, Mapo-gu, Seoul 04066, Republic of Korea; leesh9412@g.hongik.ac.kr

**Keywords:** trench field plate, vertical diode, edge termination, analytic model, breakdown voltage

## Abstract

In this study, we developed an analytic model to design a trench metal–insulator–semiconductor (MIS) field plate (FP) structure for the edge termination of a vertical GaN PN diode. The key parameters considered in the trench MIS FP structure include trench depth, MIS dielectric material and thickness, and interface charge density of MIS. The boundary conditions are defined based on the maximum allowed electric field strengths at the dielectric and semiconductor regions. The developed model was validated using TCAD simulations. As an example, a 1 kV GaN vertical PN diode was designed using the optimized FP structure, which exhibited the same breakdown voltage characteristics as an ideal one-dimensional PN diode structure without edge effects. This proposed simple analytic model offers a design guideline for the trench MIS FP for the edge termination of vertical PN diodes, enabling efficient design without the need for extensive TCAD simulations, thus saving significant time and effort.

## 1. Introduction

Gallium nitride (GaN) has emerged as a promising material for next-generation power semiconductor devices due to its superior material properties compared to conventional silicon-based power devices [1]. In particular, GaN possesses excellent material properties, including fast switching speed, high breakdown voltage, low on-resistance, and high power density [2,3]. These properties enable GaN devices to be smaller, more efficient, and to provide better performance than Si devices [4,5,6,7].

While lateral GaN devices are widely available in the market, vertical GaN devices have also received much attention to overcome the performance limitations of existing lateral GaN devices. The material quality of GaN-on-GaN wafers has continuously improved with increasing wafer size. From a processing standpoint, edge termination is crucial to reduce the on-resistance while preventing premature breakdown caused by locally enhanced electric fields at the device edge. Various edge termination techniques have been developed for Si and SiC power devices, such as junction termination extension, guard ring, field plate (FP), trench termination, floating field rings, etc. [8,9,10,11,12,13,14,15,16,17]. While ion implantation-based edge termination processes are widely used for Si and SiC power devices, they are extremely difficult to apply to a GaN device process. Limited reports are available on p-type ion implantation for GaN due to its low activation rate [18,19,20,21,22]. Moreover, p-type ion implantation requires high-temperature annealing to activate the dopants and heal the damage to the crystal structure, which poses challenges for GaN due to its low thermal stability and high thermal stress. As a result, FP or trench FP structures, rather than ion implantation-based techniques, have been employed for the edge termination of GaN power devices due to the lack of technological maturity [23,24,25,26,27]. The combination of trench metal–insulator–semiconductor (MIS) and FP structures can effectively suppress the electric field at the isolation edge of a vertical device [28,29,30,31].

While various trench MIS FPs have demonstrated excellent device characteristics for vertical GaN devices, a detailed study on design strategy for the trench MIS FP structure has not been reported. Although TCAD simulation is a powerful tool for optimizing the edge termination structure, a fundamental design strategy must be considered to determine the ranges of various structural variables such as trench depth, dielectric layer thickness, and FP length. In this study, an analytic model has been developed to optimize the trench MIS FP structure of a PN vertical diode. Three key boundary-conditions considered to design the trench MIS FP structure are (i) punch-through under the trenched area, (ii) corner electric field in the GaN drift region under the FP edge, and (iii) dielectric breakdown in MIS. The structural input variables are trench depth, dielectric material and thickness, interface charge density of MIS, and FP length. The analytic model was validated through TCAD simulation. The developed model can be utilized not only for GaN but also for other semiconductors, especially wide bandgap semiconductors where the dielectric breakdown of MIS must also be carefully taken into account due to the high critical electric field of wide bandgap semiconductors. Furthermore, the proposed model offers a useful tool for designing and optimizing the trench MIS FP structure in a time- and cost-efficient manner.

## 2. Device Structure

**Figure 1:**
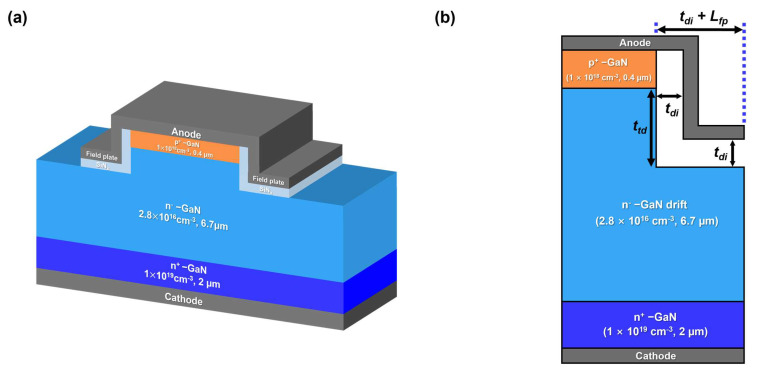
(**a**) Cross−sectional schematic of a vertical GaN PN diode with a trench MIS FP and (**b**) structural variables of trench MIS FP; $t_{td}$ is the trench depth from the PN junction, $t_{di}$ is the dielectric layer thickness, and $L_{\mathrm{fp}}$ is the lateral extension of FP.

Figure 1a illustrates the cross-sectional schematic of a vertical GaN PN diode with a trench MIS FP. The epitaxial structure consists of a highly doped p-type layer, a low-doped n-type drift layer, and a highly doped n-type contact layer. The doping concentration and thickness of the n-type drift layer must be designed to achieve a target breakdown voltage. In this study, a target breakdown voltage of >1 kV was used as an example. The doping concentration and thickness of the bottom N^+^ contact layer were set to be 1 × 10^19^ cm^−3^ and 2 µm, respectively. It is important to note that the thickness of the bottom contact layer does not significantly affect the breakdown voltage unless it is extremely thin.

Table 1 presents the thicknesses and doping concentrations of the epitaxial structure used in this study. The top P^+^ layer had a doping concentration of 1 × 10^18^ cm^−3^ and a thickness of 0.4 µm. It is known that a typical highly doped p-type GaN layer has an electrical doping concentration of low 10^18^ cm^−3^ due to the incomplete activation issue [32,33,34,35,36]. Therefore, the thickness of the top P^+^ layer must be carefully chosen to avoid punch-through at the breakdown condition. The doping concentration and thickness of the low-doped N^−^ drift layer were 2.8 × 10^16^ cm^−3^ and 6.7 µm, respectively, to achieve the target breakdown voltage of 1 kV.

Figure 1b presents the detailed structural variables of the trench MIS FP. The trench depth ($t_{td}$) is defined as the etch depth of the drift layer from the PN junction. The dielectric layer thicknesses ($t_{di}$) on the sidewall and on the trenched surface were defined to be the same. The lateral extension of the FP is denoted as “$t_{di}$ + $L_{\mathrm{fp}}$”. It was reported that the breakdown voltage reaches saturation when the lateral extension of the field plate (FP) exceeds the depletion extension along the direction of the FP [37]. In this study, the lateral extension $L_{\mathrm{fp}}$ was set to be 4.5 µm. We did not investigate the effects of the lateral extension of the FP in this study, as it does not have a significant impact as long as it is longer than the depletion extension.

## 3. Boundary Conditions of Trench MIS FP

Without a proper edge termination structure, the electric field at the edge of the active region is locally enhanced, initiating the breakdown process. To prevent premature breakdown, the electric field at the edge of the active region must not exceed that inside the active PN region. Three boundary conditions must be considered when designing the trench MIS FP structure: (i) punch-through under the MIS FP, (ii) breakdown at the trenched GaN surface under the corner of MIS FP, and (iii) MIS dielectric breakdown.

### 3.1. Punch-Through under MIS FP

**Figure 2:**
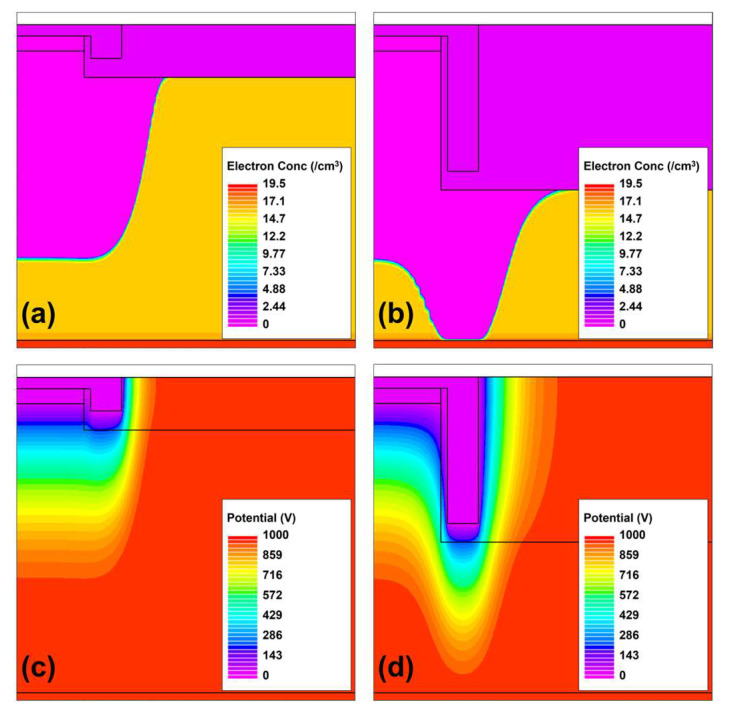
Depletion profiles (**a**) without and (**b**) with the punch-through phenomenon and their potential distributions (**c**) corresponding to (**a**) and (**d**) corresponding to (**b**).

The punch-through phenomenon under the trenched MIS FP region must be avoided until breakdown occurs in the active PN diode region. As illustrated in Figure 2, the punch-through phenomenon can be influenced through the design of the trenched MIS structure. Properly designed trenched MIS FP structures exhibit a depletion region under the trenched MIS region that does not exceed that in the active PN region, as shown in Figure 2a,c. Conversely, when the depletion region under the trenched MIS FP is deeper than that in the active PN region, the device breakdown is caused by the punch-through phenomenon under the trenched region, as shown in Figure 2b,d. Therefore, to maximize the breakdown voltage, it is essential to avoid the punch-through phenomenon under the trenched MIS region, leading to the following boundary condition: “The surface potential under the trenched MIS FP region must not exceed the potential at the same depth in the PN active region”.

### 3.2. Avalanche Breakdown at the Surface under FP Edge

**Figure 3:**
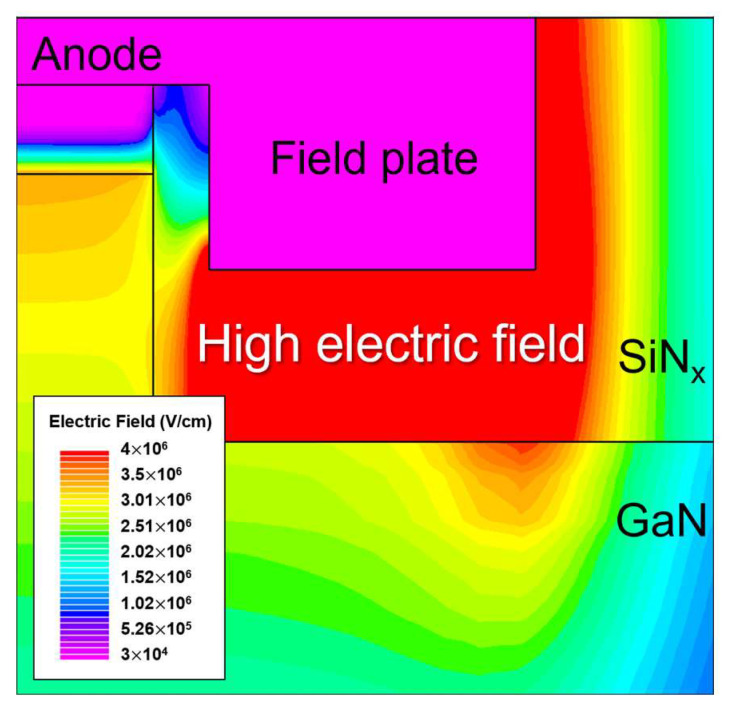
Example of electric field distribution in which the highest electric field occurs at the trenched GaN surface under the MIS FP edge.

The second boundary condition is as follows: “The electric field at the trenched surface under the MIS FP edge must be lower than the critical electric field of GaN with a sufficient safety margin”. As depicted in Figure 3, the highest electric field in the drift region under the trenched MIS FP occurs at the location under the MIS FP edge due to edge effects [38,39,40,41,42]. This enhanced electric field must not cause avalanche breakdown before breakdown occurs in the active PN region. Consequently, the electric field at the corner must be lower than the critical electric field of GaN, with an appropriate design margin. The electric field at the corner can be calculated using the image charge method [42].

### 3.3. Dielectric Breakdown of MIS Region

Due to the high critical electric field of GaN, the dielectric breakdown of the MIS region must be carefully considered when determining the thickness of the MIS layer. The electric field strength inside the MIS region is determined via the relative permittivity of the insulator material and its thickness, which can be calculated using Gauss’ law [8,9,37,38]. The third boundary condition is as follows: “The electric field inside the MIS layer must be lower than the critical electric field of the insulator with a sufficient safety margin”. From a reliability perspective, an appropriate design margin must be applied.

## 4. Analytic Model

### 4.1. Design Approach for Optimum Trench MIS FP

**Figure 4:**
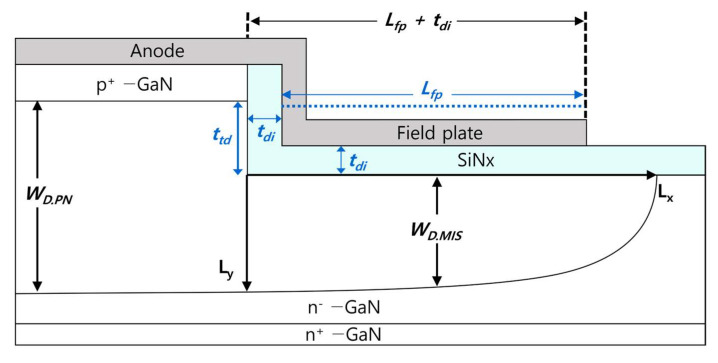
Cross−sectional structure of a vertical GaN PN diode with a trench MIS FP.

In this section, we present an analytic model for optimizing the trench MIS FP structure. Figure 4 shows the cross-sectional structure of the vertical GaN PN diode with a trench MIS FP. As discussed in Section 3.1, the surface potential under the trenched MIS region must not exceed that under the active PN region. This boundary condition leads to the following relationship:

(1) $$W_{D.PN}\;\geq W_{D.MIS}+t_{td}$$

where $W_{D.PN}$ is the maximum depletion width of the active PN diode region in the breakdown condition, $W_{D.MIS}$ is the maximum depletion width of the GaN drift layer under the trenched MIS FP region, and $t_{td}$ is the trench depth from the PN junction. Since the depletion width of each region can be expressed using Poisson’s equation, the above expression can be rewritten as:

(2) $$t_{td}\leq W_{D.PN}-W_{D.MIS}=\frac{\varepsilon_{s}E_{crit}}{q\cdot N_{D}}-\sqrt{\frac{2\varepsilon_{s}\left(V_{s}+V_{bi}\right)}{q\cdot N_{D}}}$$

where $\varepsilon_{s}$ is the permittivity of GaN, $E_{crit}$ is the critical field of GaN, q is the electric charge, $N_{D}$ is the donor doping concentration of the N^−^ GaN drift region, $V_{s}$ is the surface potential of the trenched surface under MIS FP, and $V_{bi}$ is the built-in potential of the PN diode. The above relationship can be rewritten as:

(3) $$2\varepsilon_{s}\left(V_{s}+V_{bi}\right)\leq {\left(\frac{\varepsilon_{s}E_{crit}}{q\cdot N_{D}}-t_{td}\right)}^{2}\cdot q\cdot N_{D}$$

As a result, the surface potential of the trenched MIS region derived from the above equation is given by:

(4) $$V_{s}\leq \frac{{\left(\frac{\varepsilon_{s}E_{crit}}{q\cdot N_{D}}-t_{td}\right)}^{2}\cdot q\cdot N_{D}}{2\varepsilon_{s}}-V_{bi}\approx \frac{{\left(\frac{\varepsilon_{s}E_{crit}}{q\cdot N_{D}}-t_{td}\right)}^{2}\cdot q\cdot N_{D}}{2\varepsilon_{s}}$$

The value of $V_{bi}$ is relatively small and can be ignored when $V_{s}$ >> ${\;V}_{bi}$. This first boundary condition helps avoid the punch-through phenomenon under the trenched MIS region.

In the MIS region, the electric field distribution across the dielectric layer is determined using Gauss’s law:

(5) $$\nabla \cdot \vec{E}=\frac{\rho }{\varepsilon_{0}}$$

where the $\nabla \cdot \vec{E}$ is the divergence of the electric field, ρ is the charge density, and $\varepsilon_{0}$ is the permittivity of free space. The one-dimensional application of Gauss’s law to calculate the electric field can be expressed as:

(6) $$\frac{dE}{dy}=\frac{\rho }{\varepsilon_{0}}$$

(7) ∫ε dE=∫ρ dy

(8) $$\varepsilon_{di}E_{di}=\varepsilon_{s}E_{s}=W_{D.MIS}\cdot q\cdot N_{D}$$

where $\varepsilon_{di}$ is the permittivity of the dielectric layer, $E_{di}$ is the electric field across the dielectric layer of the MIS region, and $E_{s}$ is the electric field in the depletion region of GaN. The electric field across the dielectric layer of the MIS region under breakdown conditions can be expressed as:

(9) $$E_{di}=\frac{W_{D.MIS}\cdot q\cdot N_{D}}{\varepsilon_{di}}=\frac{V_{di}}{t_{di}}=\frac{V_{Br}-V_{s}}{t_{di}}$$

where $V_{Br}$ is the breakdown voltage. According to Equation (2), the dielectric layer thickness ($t_{di}$) must be determined as a function of the trench depth ($t_{td}$) using the following relationship:

(10) $$t_{di}\geq \frac{\left(V_{Br}-V_{s}\right)\cdot \varepsilon_{di}}{\left(\frac{\varepsilon_{s}\cdot E_{crit}}{q\cdot N_{D}}-t_{td}\right)\cdot q{\cdot N}_{D}}=f\left(t_{td}\right)$$

### 4.2. Interface Charge Effects

**Figure 5:**
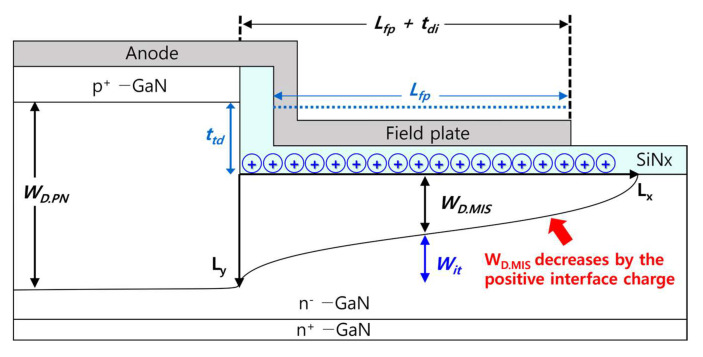
Cross-section view of the vertical GaN PN diode with the positive interface charge between SiNx.

As illustrated in Figure 5, when interface charges exist at the MIS interface, the depletion width is affected by the type and density of the interface charges. In this study, positive interface charges were assumed, but the case with negative interface charges can be derived by simply changing the sign. The change in depletion width caused by the interface charge can be expressed as:

(11) $$Q_{it}=q\cdot N_{D}\cdot W_{it}$$

where $Q_{it}$ is the interface charge density in [/cm^2^] and $W_{it}$ is the depletion width induced by the interface charges at the MIS interface. For example, when positive interface charges exist, the depletion width under the MIS interface decreases by $W_{D.MIS}-W_{it}$. As a result, the surface potential at the trenched MIS region decreases, and it can be expressed as:

(12) $$V_{s}=\;\frac{{\left(\frac{\varepsilon_{s}E_{crit}}{q\cdot N_{D}}-t_{td}-W_{it}\right)}^{2}\cdot q\cdot N_{D}}{2\varepsilon_{s}}-{\;V}_{bi}$$

As a result, Equation (10) changes to:

(13) $$t_{di}\geq \frac{\left(V_{Br}-V_{s}\right)\cdot \varepsilon_{di}}{\left(\frac{\varepsilon_{s}\cdot E_{crit}}{q\cdot N_{D}}-t_{td}-\frac{Q_{it}}{q\cdot N_{D}}\right)\cdot q{\cdot N}_{D}}=f\left(t_{td}\right)$$

An important boundary condition is that the dielectric thickness must be less than the trench depth ($t_{td}$) to suppress the electric field at the PN junction. Therefore, the maximum dielectric thickness must be smaller than the trench depth ($t_{td}$).

**Figure 6:**
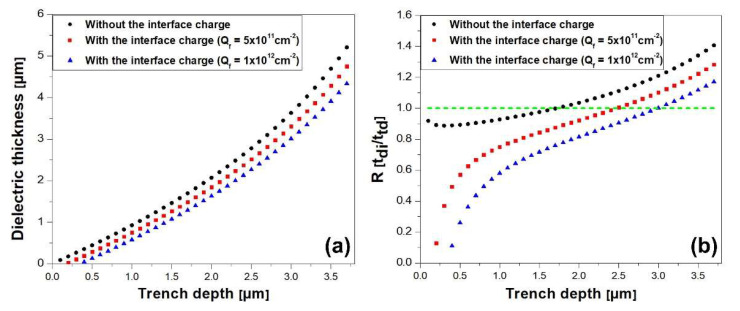
(**a**) Minimum required dielectric layer thickness versus trench depth and (**b**) ratio between minimum dielectric layer thickness and trench depth where the green line is the maximum limitation of the ratio.

Figure 6a illustrates the minimum required dielectric thickness versus trench depth as a function of the interface charge density, varied by 0, 5 × 10^11^, and 1 × 10^12^ cm^−2^. Figure 6b shows the ratio between the dielectric layer thickness and trench depth, where the maximum limitation ($t_{di}$/$t_{td}$ = 1) is indicated as a boundary condition.

### 4.3. Maximum Allowed Electric Fields in GaN Surface and Dielectric Layer

The electric field at the FP edge can be calculated using the image charge method with a uniform sheet surface charge of the FP. The electric field crowding at the semiconductor surface below the MIS FP edge depends on structural variables such as the horizontal length of the field plate ($L_{fp}$), $t_{td}$, and $t_{di}$. The electric field components in the *x*-axis and *y*-axis directions are given by [37,43]:

(14) $$E_{x}\left(z\right)=\left(\frac{qN_{D}}{2\varepsilon_{s}}\right)\frac{2W_{D.MIS}}{\pi \left(1+r\right)a}\ln\sqrt{\frac{{\left(z\right)}^{2}+{\left(1/l\right)}^{2}}{{\left(z-1\right)}^{2}+{\left(1/l\right)}^{2}}}$$

(15) $$E_{y}\left(z\right)=\left(\frac{qN_{D}}{2\varepsilon_{s}}\right)\frac{2W_{D.MIS}}{\pi \left(1+r\right)a}\left[\tan^{-1}zl-\tan^{-1}l\left(z-1\right)\right]$$

(16) $$E_{t}\left(z\right)={\left[{E_{x}}^{2}\left(z\right)+{E_{y}}^{2}\left(z\right)\right]}^{\frac{1}{2}}\;$$

where *a* = $\tanh\left[\tan^{-1}L/2W_{D.MIS}\right]$, *r* = $\varepsilon_{di}/\varepsilon_{s}$, *z* = $x/L_{fp}$ and l = $L_{fp}/t_{di}$.

**Figure 7:**
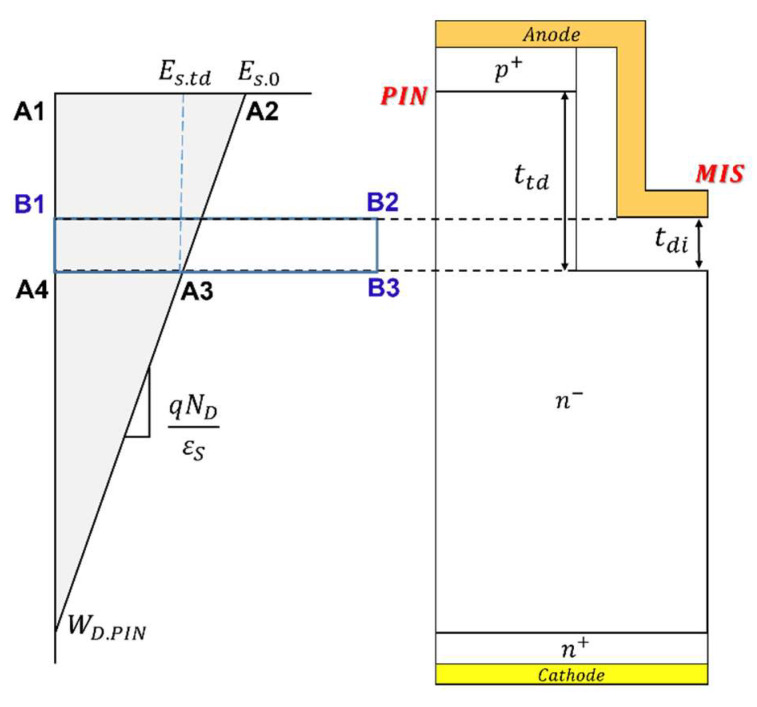
The 1D electric field distributions along A1–A4 and B1–A4.

When the trench MIS is properly designed without punch-through under the trench MIS FP, the surface potential at the trenched region must be equal to that at the same depth from the PN junction inside the active region. In such a case, the electric field inside the MIS region depends on the dielectric layer thickness. Figure 7 illustrates the electric field distribution under the PN junction in the active region and under the trenched MIS FP region. Since the same voltage is applied, the area of A1A2A3A4 is equal to the area of B1B2B3A4. Therefore, the maximum electric field in the MIS region ($E_{di}$) can be expressed as:

(17) $$E_{di}=E_{s.0}\cdot \left[1-\left(t_{td}/2W_{D.PN}\right)\right]\cdot t_{td}/t_{di}$$

**Figure 8:**
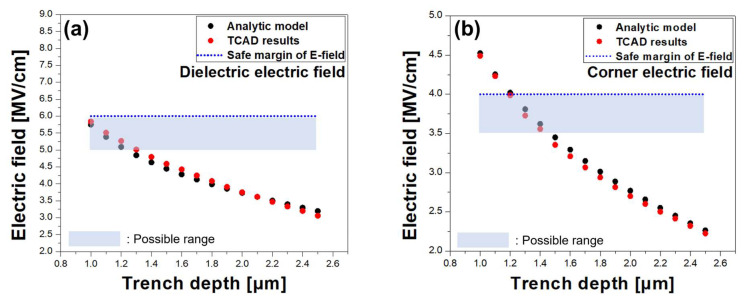
Comparison between TCAD simulation and analytic model; (**a**) maximum electric field in the dielectric layer under MIS FP. (**b**) Maximum electric field at the trenched GaN surface below the MIS FP edge. The dashed lines are the safety margin of GaN and dielectric material.

The TCAD simulations were conducted with various dimensions to validate the model. The trench depth was varied from 0.7 µm to 2.5 µm where the interface charge density was 1 × 10^12^ cm^−2^ and the minimum dielectric thickness was determined using Equation (13). Figure 8a shows a comparison between TCAD and the calculated maximum electric field at the trenched GaN surface below the MIS FP edge versus $t_{td}$ where $t_{di}$ was the minimum dielectric layer thickness needed for $t_{td}$, obtained using Equation (13). The difference between TCAD and the calculation was not significant. The boundary condition for the critical electric field of GaN must be considered to determine the safe margin of $t_{td}$. In this study, the maximum allowed electric field for GaN was set to be 4 MV/cm as an example.

Figure 8b shows a comparison between TCAD and the calculated maximum electric field in the trench MIS region. Again, agreement is observed between the TCAD simulation results and the analytic model. The maximum allowed electric field for the dielectric layer must be carefully defined for long-term reliability concerns. In this work, it was set to be 6 MV/cm for a given SiN_x_ dielectric layer. In conclusion, the trench depth ($t_{td}$) must be larger than approximately 1.1 µm based on Figure 8a,b, taking into account the maximum allowed electric field strengths for GaN and SiN_x_.

Our analytic models calculate not only the minimum dielectric thickness required for a given trench depth but also the maximum electric field strengths for GaN and the dielectric layer. This information allows one to determine the trench depth and dielectric thickness based on the boundary conditions of the maximum allowed electric field strengths for GaN and the dielectric layer.

## 5. Design of GaN PN Diode with a Trench MIS FP Structure

**Figure 9:**
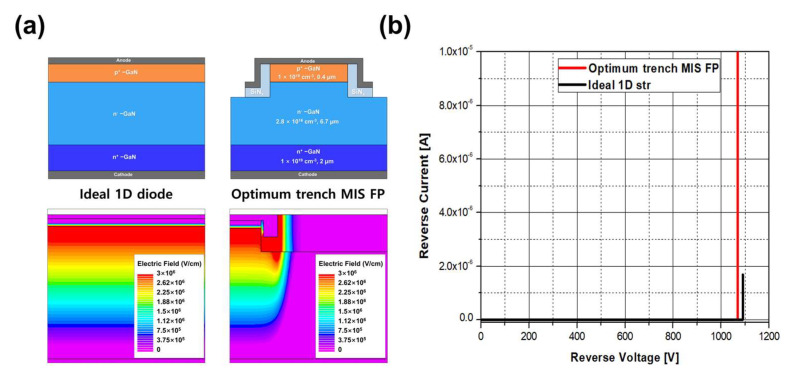
(**a**) Structures and electric field distributions of an ideal 1D vertical PN diode and a vertical PN diode with a proposed trench MIS FP, and (**b**) breakdown characteristics obtained using the proposed trench MIS FP in comparison with an ideal 1D diode structure. The electric field distributions were simulated at a reverse bias voltage of 1080 V.

The range of the trench depth can be determined based on the boundary conditions discussed above. From a fabrication perspective, it is preferable to use the minimum trench depth with a minimum dielectric layer thickness for a given trench depth. Consequently, the proposed structure was determined to have a minimum trench depth of 1.2 µm and a dielectric layer thickness of 0.77 µm. Figure 9a illustrates an ideal vertical PN diode without edge effects and a vertical PN diode with the proposed trench MIS FP structure. The doping concentration and thickness of each epitaxial layer were the same. Breakdown simulations were performed for both device structures, and the results are compared in Figure 9b. Both structures exhibited a similar breakdown voltage, approximately 1.1 kV.

**Figure 10:**
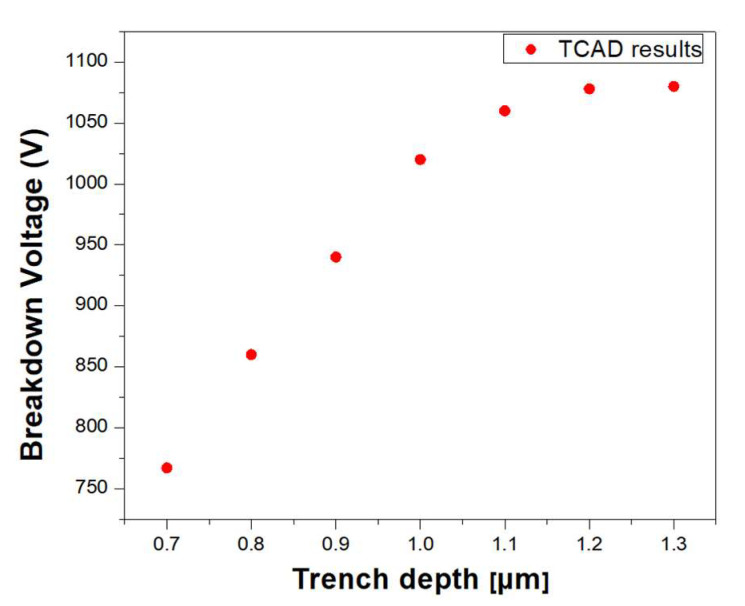
Breakdown voltage versus trench depth ($t_{td}$) obtained using TCAD simulation.

To validate the design strategy, additional TCAD simulations were conducted using smaller trench depths. Figure 10 shows the simulated breakdown voltage versus trench depth ($t_{td}$) when the minimum dielectric layer thickness defined using Equation (13) was used. The breakdown voltage decreased monotonically as $t_{td}$ decreased below 1.2 µm because the electric field at the GaN surface became higher than the maximum allowed electric field. It is important to note that the dielectric breakdown model is not included in the TCAD simulation since TCAD considers the dielectric material as a perfect insulator. Therefore, the boundary condition of the maximum allowed electric field in the MIS region must be taken into account when designing the MIS region, and the analytic model provided in this work is useful for incorporating the dielectric breakdown condition.

## 6. Conclusions

In conclusion, this study presented the design and optimization of a trench MIS FP structure for vertical GaN PN diodes. An analytical model was developed to optimize the structure and calculate the minimum dielectric layer thickness as a function of various input design parameters, such as trench depth, dielectric layer thickness, and interface charge density. The results obtained from the TCAD simulations were consistent with the predictions of the analytical model. The proposed analytical model provides a valuable tool for designing and optimizing the trench MIS FP structure in a time- and cost-effective manner.

## Figures and Tables

**Table 1:** Epitaxial structure of the vertical GaN P^+^/N^−^/N^+^ diode.

Structures	P^+^ GaN	N^−^ GaN	N^+^ GaN
Thickness (µm)	0.4	6.7	2
Doping concentration (cm^−3^)	1 × 10^18^	2.8 × 10^16^	1 × 10^19^

## Data Availability

Data sharing not applicable.
